# Students’ Perception towards New Face of Education during This Unprecedented Phase of COVID-19 Outbreak: An Empirical Study of Higher Educational Institutions in Saudi Arabia

**DOI:** 10.3390/ejihpe12070061

**Published:** 2022-07-19

**Authors:** Mohammad Asif, Mohammed Arshad Khan, Sufyan Habib

**Affiliations:** College of Administration and Financial Science, Saudi Electronic University, Riyadh 11673, Saudi Arabia; s.habib@seu.edu.sa

**Keywords:** students’ perceptions, online learning, face-to-face learning, COVID-19 pandemic, higher educational institutions

## Abstract

**Purpose:** To examine the perception of students regarding an e-learning system adopted by various educational institutions in the Kingdom of Saudi Arabia during the COVID-19 pandemic. **Methodology:** A web-based-survey was conducted among selected university students in Saudi Arabia. A total of 294 students were randomly chosen to determine the utilities and credibility of the adopted e-learning mode of education. The reliability of latent constructs was assessed according to Cronbach’s alpha, and confirmatory factors analysis was conducted via AMOS software (version 24) to measure the students’ perceptions of online learning. **Results:** The outcomes of the present study reveal that e-learning has been very useful throughout the pandemic period among selected Saudi Arabian universities. The students have a positive view of the online system of education, which has many benefits, including flexibility, low cost, self-learning, and convenience. **Implications:** The results of the present study will be beneficial for all educational institutions that are largely dependent on the findings of the online survey.

## 1. Introduction

The COVID-19 pandemic has affected many aspects of people’s lives, majorly impacting sociocultural, economical, as well as educational aspects. These impacts have been experienced by educational institutions around the globe. Students’ lives have been impacted by this pandemic from the onset, as all educational institutions had to be closed, with the whole education system shifting to the use of online platforms. Thus, online learning took place throughout the world, and numerous technologies and the internet have played an important role in program management, the creation of material, and educational distribution [1].

All educators were forced to adopt online learning, as school buildings were not open. Even though a number of people did not feel ready for this change, students had to adjust themselves to this new learning process while dealing with many other challenges resulting from this pandemic [2]. Learning shifted from an offline to online mode, with the hope that the effects on learning would be mostly beneficial. There is remarkable proof of a higher student success rate when using an online learning mode. The effectiveness of planned online learning and planned and onsite learning is indistinguishable and supported by substantial evidence [3].

Although the number of problems faced by students as well as educators during online learning is mostly related to the difficulty in using e-learning applications mainly experienced by those in remote areas, there are also issues of an increased number of assignments, disturbances due to poor network connection, and so forth. These obstacles also negatively impact students’ motivation to complete assignments. As a result of these issues, they do not perceive the optimal outcomes of learning, and thus, the objective of e-learning is not always successfully achieved [4]. There are many learners who are self-motivated, intelligent, and self-directed, and they have achieved success from the online learning mode in spite of its obstacles. The parents of some students stated that their wards became indolent when it came to studying online. This shows the negative side of the learning attitude of students with respect to the online learning mode [5].

As the pandemic altered the whole learning system, students and teachers were also trying to adapt to these recent developments in the learning process and the new mechanisms governing teaching and learning methodology. As there is a strong need for e-learning instead of conventional teaching and learning processes, as per the current scenario, it is also important to be aware of students’ opinions regarding e-learning [6]. Knowledge of the learners’ readiness for this new teaching methodology, and their ideas, if they have any, are significant when it comes to creating a considerable change and determining the degree of adaption. The objective of our survey is thus to assess the viewpoint of learners toward online classes conducted by the numerous institutions during the lockdown phase of the pandemic.

### 1.1. Background and Scope of the Study

The education sector has experienced significant evolution, shifting from a teacher- to learner-centered mode. As a result of the former, teachers play an important role as knowledge informants, while learners play a passive role as mere recipients of that knowledge [7]. However, within a learner-centered approach, learners are the producers of knowledge in the class under the guidance of the teacher. In this approach, teachers answer the assignments and motivate learners to find alternate solutions. The internet and a number of new technologies have been incredible facilitators of a learner-centered approach [8]. Online learning has been a major development in the education sector in the 21st century [9]. Online learning or e-learning can be understood as learning through the internet and various different technologies which help a student to almost immediately access information.

During the COVID-19 pandemic, face-to-face learning was not possible, and e-learning was the only solution. However, the experience of online learning varies from country to country. For some countries, it is fairly easy to facilitate online learning, whereas for some countries with middle- and low-income groups, implementation is challenging due to a lack of proper resources. In the Arab region, many countries, such as Qatar, Jordan, Bahrain, Kuwait, Emirates, and the KSA, are more developed than others [10]. Most of the higher educational institutions in Arab countries shifted to asynchronous and synchronous learning systems during the pandemic. The Arab country Jordan started e-learning according to the Education and Planning and Information Technology Ministries in 2002 [11]. Jordan also initiated an online instruction system, switching from conventional to virtual teaching. Similarly, Talal Abu-Ghazaleh University adopted an online mode for the recruitment and enrolment of new scholars and organized online classes for the first time in 2012. The University of Jordan launched synchronic “blended learning”. This is a kind of learning in which a mixed system of online and onsite learning is present, and as a result, some practical courses are conducted on a university campus while the theoretical courses are run online. Jordan is among the countries that retaliated to the calamity by launching an online platform, “Darsak”, to ease virtual learning in schools [12]. However, in Jordan, the online learning method was not adopted in schools before the pandemic.

On 11 March 2020, the World Health Organization (WHO) declared COVID-19 a global epidemic, and on 19 March, it was declared an emergency toward preventing the spread of disease. As an effect of this, curfews were imposed around the world for approximately two months, with universities being closed for longer periods. Consequently, face-to-face education was not possible, but learning discontinuity was also not acceptable, and therefore, the need for online education arose to ensure the maintenance of social distancing. Online education or learning has been a vital mechanism for today’s learning, since it provides students with access to an education platform at all times so that they can conveniently learn in a place and at times suitable to them. This facilitates learning with flexibility independently of time and place [13]. Online learning also provides answers to questions and helpful feedback on the contents of assigned courses to learners [14].

### 1.2. Literature Review and Gap Identification

The evolution of information and communication technology has added numerous advantages to the lives of humans. Now it is important not only to be aware of the technology but to have a better understanding of it [15]. Because of the development of e-technology and better internet networks, the whole platform has changed from an onsite to online learning mode [16]. On top of the different electronic media such as television, satellite, and CD-ROM, e-learning is classified in the following way: “education delivered via internet”, and virtual learning is referred to as “education delivered only via the internet or web-based media” by some experts [17]. E-learning is stated as “bridging the space between teachers and students through the use of web-based technology” [18].

The internet has been very useful for humans in numerous ways, mainly in the area of education. In recent times, new techniques of learning have depended on the use of technology [19]. However, not every student can accept and adapt to this change. Each and every student is different from others in many aspects such as age, ability of thinking, and interest in technology, and these attributes affect the acceptance of change in the process of learning [20]. The reactions of students to online learning differ according to their ages, with the older students manifesting more acknowledgment for online learning. There are still some crucial divergences in learners’ views of online learning. There is concern for the effectiveness of e-learning environments [21].

The seriousness of students in online learning can be judged by their activeness in engaging in learning. The three aspects essentially required to be engaged in online learning are emotional, behavioral, and cognitive involvement [22]. These aspects are described as follows: (1) Behavioral participation—participation shown by the actions of heeding to learning during online class. (2) Cognitive participation—participation where a learner obtains a set of skills in the online learning mode. (3) Emotional participation—participation through which the learner has positive emotions for online learning, the teacher, as well as peers. The advancement of online learning in present times is the reason for the growth in the number of online courses conducted by colleges and school [23]. In addition to this, students’ demand and technological evolution for online classes has prompted colleges and universities to execute online courses with normal learning [24]. A notable point is that online learning is not yet a requirement in schools, though it is considered as an advanced technique for dealing with challenges encountered in the learning process [25]. Now, many universities are implementing plans to invest in modern classes and in training and recruitment in faculties to teach online [26]. A survey indicates that online learning is expected to grow more remarkably in educational institutions and in corporate organizations in the near future [27]. It is supposed that online learning is interactive because of these recent developments, and online education provides students with an environment where they can actively engage with the content and practically learn, and it also increases understanding as they attain new knowledge [28]. Furthermore, online learning has been more important in the past few years globally, shifting the idea associated with universities that “online class is optional” to “online class is necessary” [29].

Thus, on the basis of the above-discussed extensive review of the literature, it seems that although studies have been carried out to explore students’ perceptions of online learning, the interpretation of students’ views on online learning in the context of the COVID-19 pandemic has not been addressed.

### 1.3. Statement of the Problem

The ultimate aim of conducting this study was to analyze students’ perceptions of online learning. The present study was centered on those colleges and universities that are providing education to their students via online means. In today’s environment, one of the most critical challenges for universities is to engage students with study through their e-learning system. The study was carried out from the perspective of students enrolled in various universities and colleges in the KSA. Therefore, it was pertinent to examine students’ perceptions of online learning. The present study attempted to answer the following research questions:

1. How do the students enrolled in various universities in Saudi Arabia perceive the importance of an e-learning system during the lockdown phase of the COVID-19 pandemic?
2. What are the benefits of the e-learning method of education undertaken by the various universities in the KSA from the perspective of students?
3. How does the online learning system influence the students’ attitude toward e-learning adopted by numerous educational institutions during the pandemic in the KSA?

### 1.4. Objectives of the Study

The main objectives of this study were as follows:

1. To study the perception of students regarding the significance of an online mode of learning during the lockdown phase of the COVID-19 pandemic in the KSA.
2. To examine the benefits of an online learning system from the learner’s point of view in Saudi Arabia.
3. To analyze the attitude of the students toward the e-learning system adopted by the colleges and universities in Saudi Arabia.

## 2. Method

Research methodology is the specific procedures or techniques used to identify, select, process, and analyze information about a topic. In the present article, the methodology section is designed in such a way to allow readers to critically evaluate the study’s overall validity and reliability.

### 2.1. Participants of the Study

In this study, the population was students who were undergraduates and postgraduates. The sample comprised 294 students randomly chosen from the various universities and colleges in the Kingdom of Saudi Arabia (KSA), and they provided important feedback on their perceptions of the online learning mode.

### 2.2. Development of the Survey Instrument

A survey method was used in the study for the purpose of primary data collection. This study was conducted on the basis of an online survey to maintain the various protocols of the pandemic recommended by the government. The survey was conducted during the period of May–October 2021, when all the educational institutes in the KSA were closed due to widespread cases of COVID-19. For this study, the web-based survey was created with the help of Google Forms, and a link to the survey together with an informative description of the study and invitation to the survey was sent out to students enrolled in numerous colleges and universities in the KSA. We mentioned the core objective of the study in the survey instrument, i.e., *“First, we asked the participants to take part in a research study, and the main aim behind conducting this research is to elicit the feedback of the university students at large regarding their perceptions of e-learning and the effectiveness of online sessions amid the COVID-19 lockdown phase. We sincerely request your active participation in this survey. The information collected through this survey will be exclusively used for academic purposes only and be kept strictly confidential.”* To summarize, we collected an informed consent letter from each of the students for this study, with the necessary details of all the investigators involved in the present study being shared along with the purpose, study procedures, and risks and benefits associated with the study, and the ethical standards of research were also respected. This online survey comprised two sections: Section A gathered students’ demographic information, while Section B ascertained the perceptions of students regarding the online learning system in the course of the COVID-19 pandemic. This survey was based on answers corresponding to a five-point Likert scale ranging from strongly disagree (1) to strongly agree (5). For the validity and reliability testing of the survey instrument, the modified questionnaire was evaluated by experts, a pilot survey was conducted among 84 students, and appropriate changes were accordingly incorporated prior to its final circulation to the target population. On account of reliability, Cronbach’s alpha was used in the present study to specify how the items were precisely correlated. 

### 2.3. Sampling Procedure and Data Analysis

In order to determine the appropriate sample size, the rule of thumb adopted by various authors in the past studies was applied [30]. The rule of thumb states that the sample size should be five times the number of statements used in the study [31,32,33,34]. Accordingly, 30 statements were used by the researchers in the study, and the adequate sample size for the present study was therefore determined to be 30 × 5 = 150. The researchers collected primary data from 294 sample students enrolled in various universities and colleges located in the KSA [35]. Therefore, we can say that the sample size was adequate and also representative of the population for the current study [36].

The collected sample dataset was imported into SPSS (v-25) software through Microsoft Excel to allow analysis by the researchers [37]. Confirmatory factor analysis (CFA) was conducted in this research using AMOS (v-24) software to check whether all the studied variables were empathetically interpreted based on their relevant latent construct. Researchers have applied numerous statistical tools and methods such as KMO, Bartlett’s test, and descriptive statistics (including mean and standard deviation), among others, using SPSS (version 25) software to study the advantages and students’ perceptions of online learning throughout the pandemic [38].

## 3. Results

The findings from the qualitative and quantitative analysis of the sample dataset gathered for the present study are depicted below.

### 3.1. Demographic Information of the Sample Respondents

Basic information of the university students is presented in this section. Table 1 depicts the outputs of the questionnaire concerning age, gender, course, and level of education. The data shown in this table are compiled from the primary data.

Represented in the above table are the demographic details of the participants categorized on the basis of their age, gender, study level, course of study, their institution, and their present status. It shows that most sample respondents (58.5%) were males, while 41.5% were females. In addition, the aforementioned data indicate that students (75.2%) were mostly from the age group of up to 30 years, 24.1% belonged to the group of 21–25 years, and the smallest group of 0.7% of students/participants fell within the age bracket of over 50 years. The research outcomes reflect the youthful views of the majority age group, i.e., up to 30 years.

Participants were classified on the basis on their study courses. As Table 1 indicates, out of 294 participants, 87.7% of the participants were from business stream in general and accounting and marketing in particular. The unbiased presentation from the other courses of study is as follows: Art and Design, 6.5%, IT, 2.4%, and Engineering, 3.4%.

Moreover, the data gathered on sample participants were fairly diffuse on the basis of their academic year. The majority of students (33%) were in the second year, followed by third-year students (31.6%), then first year students (19%), and the remaining 16.3% of students were in the fourth year. As a consequence, this research embraced diversified groups of learners to obtain a better representation of views.

Furthermore, the majority of learners were associated with Saudi Electronic University, i.e., 18%, followed by students of Dar Al Uloom University (13.6%); 13.3% were from Prince Sultan University, 11.6% belonged to Shaqra University, 10.9% were studying at King Abdulaziz University, 10.5% belonged to Al Yamamah University, and the very least, i.e., 10.2%, were from the Alfaisal University.

### 3.2. Reliability of the Latent Constructs

According to Cleff (2014), Cronbach’s alpha is the standard measure of internal correspondence between items in a scale. The fundamental objective of reliability testing was to examine the attributes of the scales of measurement and the items for getting the overall index of internal consistency of the scales. The conclusions of the test are mentioned in the following table.

Values of Cronbach’s alpha between ±0.41 and ±0.70 indicate the moderate reliability of the measured scale, while values exceeding ±0.70 indicate high internal consistency. Shown in the table are the results of the fidelity analysis of latent constructs used in the research study. Because the values of Cronbach’s alpha surpassed the threshold limit, it can be interpreted there was high internal reliability (Chan and Idris, 2017). Table 2 of the reliability analysis further affirms that the coefficient alpha of each latent construct was greater than the approved limit, i.e., 0.70, indicating the existence of strong internal consistency between the items in the scale chosen for the study.

### 3.3. Exploratory Factor Analysis (EFA)

EFA is a conventional approach for scale refinement. It comprises the following steps: (1) identifying the items relevant to a particular domain from the literature, (2) designing a survey instrument to measure these items, (3) conducting a field survey, and (4) applying EFA (frequently with varimax rotation) on the manifested variables. Subsequently, major factors are identified according to the factor loadings of various items. This is a data-driven approach to extract underlying factors or latent variables from a set of measured variables. Accordingly, EFA was first conducted on 14 items of Students’ Perceptions of Online Learning to group the variables. These items were analyzed through principal component analysis (PCA) using SPSS (version 25). Prior to performing PCA, the suitability of the collected sample dataset was assessed for factor analysis. Firstly, the KMO measure of sampling adequacy was applied by the researchers to assess the appropriateness of using factor analysis on the dataset.

#### 3.3.1. KMO (Kaiser–Meyer–Olkin) and Bartlett’s Test of Sampling Adequacy

Adequacy of sampling was tested using the KMO and Bartlett’s tests, which also determined the necessity of conducting factor analysis. Factor analysis was performed after a positive KMO and Bartlett’s test. The results of the KMO and Bartlett’s test are depicted in Table 3 below:

As per Table 3, the Bartlett test of sphericity was significant with *p* < 0.001, which was less than the alpha level of 5%, and the KMO measure of sampling adequacy was 0.959, which was far higher than the threshold limit of 0.6. The results in Table 3 reveal that the data are suitable for structure detection. The high value of KMO (i.e., KMO > 0.6) and small value in Bartlett’s test, i.e., less than the 5% significance level, indicated that factor analysis was useful with the sample dataset collected from the students of universities situated in Saudi Arabia.

#### 3.3.2. Exploratory Factor Analysis: Students’ Perceptions of Online Learning

Table 4 depicts the categorization of the latent construct, namely “Perception of Students toward Online Learning”, into four extract components on the basis of their factor loadings using the rotated component matrix.

Table 4 shows that UEL1, UEL2, UEL3, and UEL4 were grouped as Factor-1, i.e., Perceived Usefulness of E-learning (PUEL); SEL5, SEL6, and SEL7 were grouped as Factor-2, i.e., Perceived Self-Efficacy of Using E-learning (PSEL); EEL8, EEL9, EEL10, and EEL11 were grouped as Factor-3, i.e., Perceived Ease of Use of E-learning (PEEL); and IEL12, IEL13, and IEL14 were grouped as Factor-4, i.e., Behavioral Intention of Using E-learning (BIEL).

### 3.4. Confirmatory Factor Analysis: Students’ Perceptions of Online Learning

**Figure 1:**
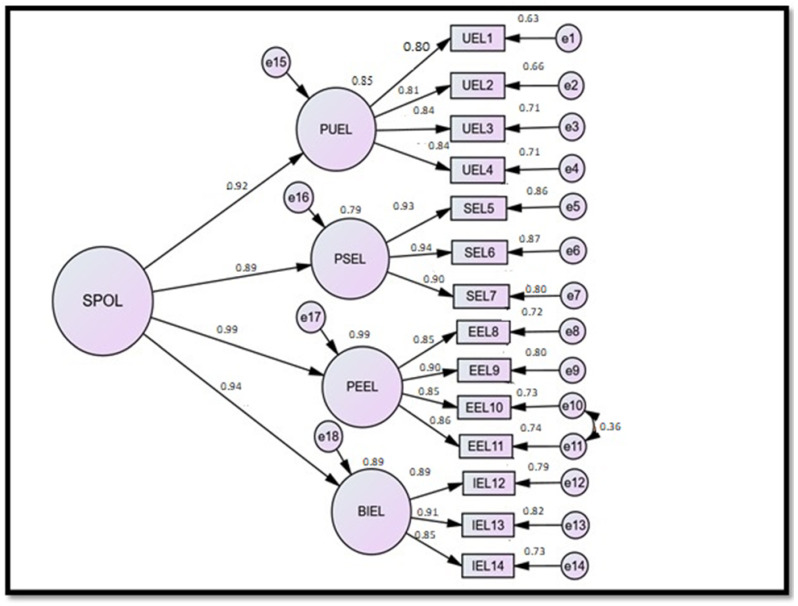
CFA measurement model for Students’ Perceptions of Online Learning.

The researchers used confirmatory factor analysis (CFA) in this study through AMOS (version 24) software to check if all the apparent variables distinctly explained their relevant latent construct. In consonance with this research study, intending to analyze the students/learners’ perceptions of the online learning system, the main latent construct named “Students’ Perceptions of Online Learning (SPOL)” was classified into four subconstructs, and additionally, these subconstructs were measured using different statements selected by the researchers to gather feedback from the survey participants. It is displayed in Figure 1.

Figure 1 showcases that the principal latent variable, namely “Perception of Students toward Online Learning”, was computed using its four subconstructs: “Perceived Usefulness of E-learning (PUEL), Perceived Self-Efficacy of Using E-learning (PSEL), Perceived Ease of Use of E-learning (PEEL), and Behavioral Intention of Using E-learning (BIEL).The first subconstruct (PUEL) was computed via four statements (UEL1, UEL2, UEL3, and UEL3) expressed by small rectangular boxes. The second subconstruct (PSEL) was computed via three items coded as SEL5, SEL6, and SEL7. The third subconstruct (PEEL) was measured via four items coded as EEL8, EEL9, EEL10, and EEL11. The last subconstruct (BIEL) was computed via three statements coded as IEL12, IEL13, and IEL14.

The fraction of unexplained variation is indicated by the small ‘e’. The standardized regression coefficient for a specific item is indicated close to the arrow leading to the particular item, whereas the value above each corresponding item manifests as the “squared multiple correlations (R^2^)” or the explained variation in the computed variables. Table 5, Table 6 and Table 7 depict the analysis summary of the CFA measurement model provided by AMOS (version 24).

The above table shows the Chi-square (χ^2^) value, i.e., 0.162, which was more than 5%, and the CMIN/DF value of 2.791, which was not as high as the recommended limit, i.e., 3. Thus, these values show that the collected sample dataset was suitable for the model fit. Values for a further four goodness-of-fit indices were determined, namely GFI = 0.907, AGFI = 0.866, CFI = 0.969, and NFI = 0.953. These values exceeded their threshold limits, indicating that the CFA measurement model for “Students’ Perceptions of Online Learning” was a well-fitting model. Values for the two badness-of-fit indices, that is, RMSEA = 0.039 and SRMR = 0.054, were both below the approved limits, indicating that the accumulated sample dataset fit the model appropriately. Hence, the CFA measurement model was found to be an appropriate model.

Table 6 shows that the computed variables were remarkable with relation to their proportional constructs, because their *p*-values were below the approved limit of 5% alpha level. In addition, the standardized regression weight (β) of each path was above 0.40, which attested to the convergent validity of the previously discussed and attained CFA measurement model, which also showed that each apparent variable was highly correlated with its respective latent construct (Tarka, 2018). The critical ratio (CR) value is the statistics formed by dividing an estimate by its standard error. The CR values were more than the recommended limit of 1.96 for the regression weights, leading to the conclusion that each path was significant at the 5% significance level or higher (i.e., the estimated path parameter was significant).

Table 7 shows that the composite reliability value of each and every variable was more than the threshold limit of 0.70, indicating that strong internal consistency existed among the items in the scale. On the other hand, the average variance extracted for each latent construct exceeded the approved limit of 0.50. This confirmed the strong convergent validity of the measurement model, as discussed.

This section of the survey was also used to enquire about students’ perceptions of the online learning mode adopted by the colleges and universities in Saudi Arabia amidst the COVID pandemic. The sample respondents (students) were asked to provide their opinions according to a five-point Likert scale ranging from strongly disagree (1) to strongly agree (5). The respondents recorded assorted observations of online learning (e-learning). This rating was classified into three categories: First and foremost, the mean score of 3 depicted the equitable feedback of respondents. Second, a score above the 3 showed students’ positive perception for e-learning. Finally, a mean value below 3 represented the students’ negative attitude toward the online learning and education system. Table 8 represents the results of this section.

Table 8 explores the efficacy of online learning from the students’ perspectives. The efficacy of online learning encompasses providing learning during the crisis with mean values of greater than 3. This means the study respondents perceived that online learning is useful during the lockdown phase of the ongoing outbreak. The sample respondents showed that studying using an online mode of learning provides flexibility for them with respect to time and space. They also showed their agreement on e-learning enabling interactive communication with their educators without face-to-face meetings.

Students opined that they feel confident while using and operating the online learning platforms. They feel confident while accessing the online learning content, since it facilitates them in gaining new experiences and skills, with the mean value of 3.73 and standard deviation of less than 0.983. The study respondents perceived that the various platforms using online learning systems are user-friendly, with a mean value of more than 3 and standard deviation of 0.120. By using online learning platforms, students are able to get necessary and updated information. Moreover, they added that e-learning platforms can ease the method of learning, and even the setup of the same is suitable for students’ way of acquiring knowledge and skills. Thus, we can conclude that online learning motivates learners to participate in the learning process, as the manner of instruction has been transformed to self-paced learning in which the focus is completely on the student.

Finally, the findings listed in the aforesaid table show that learners make use of online learning as a support system for their learning and updating their knowledge on various subjects and keeping track of amendments, if any. They also believed that e-learning is a free-learning tool for gaining new and up-to-date information pertaining to different kinds of fields, whatever the type of information they want to obtain. This is because the mean score of the statements related to “Behavioral Intention of Using E-learning” was more than 3, and the value of standard deviation was below one. Therefore, we can conclude from the above analysis that the behavioral intention of students toward e-learning is pragmatic, positive, and motivating. Thus, it can be anticipated that students’ perceptions of using interactive e-online learning would positively influence their contentment.

### 3.5. Benefits of Online Learning

This study also explored the efficacy of online learning throughout the COVID pandemic. According to the participants in this study, online learning is advantageous to them on account of the numerous reasons discussed in Table 9. To compute the degree of benefits from online learning, various questions were asked, and answers were rated according to five-point-Likert-scale-based statements ranging from strongly disagree to strongly agree. The results of this section are represented in the table below.

It is evident from Table 9 that the research sample attested to the effectiveness of online learning with mean scores of more than 3 and values of standard deviation below 1. The survey participants suggested that online learning ensures that they will have access to content according to a time and place that is suitable for them if online classes are asynchronously recorded and accessible around the clock. Students also expressed that they can easily and expeditiously share educational material with their educators and friends. The participants also showed their agreement on collaboration work and the issue of interactivity among students with the mean value of 3.20 and a value of standard deviation of 1.214.

Furthermore, online classes assist learners in participating in various courses of higher education no matter if they take the classes “asynchronously” (recording the study) or “synchronously” (the exact time of the lecture). Learners emphasized their satisfaction with the working of the online learning system and their interaction with the educators/teachers during online teaching and learning as well. Thus, it can be seen that most of the learners have the potentiality to leverage the online learning mode as they find it easy to use.

Online learning has accumulated various types of learning methods. The survey participants also opined that a system that has many tools implanted in it to make it conjunctive and manageable for an online course is an efficient learning management system, with the mean score of 3.60. The variable “quick feedback” received positive responses from the majority of students. Especially in connection with online learning, instant response is necessary because timely and instant feedback keeps students engaged and entailed in discussion and thus contributes to the online learning process.

The overall number of positive answers about “wide and diverse interactions” revealed that the respondents are learning in an Open and Distance Learning (ODL) context because they know that interaction is always there, even if they are not in the institutes. This also shows that the respondents are aware of the good space created by the cultural diversification among them, which is also available on the online interaction platform during the learning process.

The responses of the sample students included in the survey regarding “updated learning material” were positive. Updated learning material in an e-learning environment has been taken as a positive thing. Updated learning material keeps up the standard of the course, which is internationally recognized, and is also an advantage. With reference to “access study resources effectively”, the outcomes showed that the e-learning platform also assists students in effectively acquiring numerous study resources, with a mean score of 3.83 and standard deviation of 0.867.

### 3.6. Attitude of Students toward Online Learning

The researchers sought to investigate students’ attitudes toward online learning. The following questions were also answered on the basis of a five-point Likert scale corresponding to statements ranging from strongly disagree to strongly agree. The categories strongly agree and agree were merged to the positive answer, i.e., agree. The corresponding process was used to merge strongly disagree and disagree to attain a single answer from the respondents, i.e., disagree. The option “neutral” was not altered. The outcomes are shown in Table 10.

The results of the aforesaid analysis in Table 10 show that 64.7% of the respondents were in favor of the idea of online learning adopted by educational institutions during the pandemic. Around 19% of students were neutral, and only 16.3% students were not in the favor of online learning system. Furthermore, a majority of the students (68.1%) opined that online learning is a very innovative system for acquiring knowledge and skills, 18.7% of the students were undecided, and 13.2% did not agree with this.

The findings of the present study also reveal that 56.4% of the sample respondents believed that classes conducted by the institutions through online learning platforms will be very interesting, 17.7% of the students disagreed, and 25.9% were neutral. On the other hand, 58.9% of the sample students also felt that the online mode of learning was not suitable for practical demonstrations, while 17% students indicated that online learning is acceptable in the case of courses that required practical classes, and 24.1% of the students were undecided.

In addition to the above, only 20.4% of the survey participants felt that the employers of companies gave preference to students who had conducted their studies through an electronic mode of learning. In this matter, 55.8% of students opined that the companies gave priority to those students/learners who have completed their education in the traditional mode of classroom learning. As far as the cost of online learning is concerned, 57.6% of the students agreed that conventional classroom learning is more expensive than e-learning, while 24.1% of the respondents were neutral, and only 18.3% students believed that the online mode of learning is costlier than the traditional mode of learning.

## 4. Discussion

Online learning provides a platform for learners to learn in a unique and different way. Education has also been improved by the use of online education platforms. Teachers and students both have optimistic views regarding online platforms, although many improvements in online learning are still needed. Thus, it can be demonstrated that there are many benefits of online learning, and moreover, it has increased the rate of literacy by spreading to each and every corner of the globe. With efforts to prevent the spread of the novel coronavirus, the contours of the education system are changing, with online education becoming the primary means of instruction. Universities and institutions are shifting to online platforms to catch up with the curriculum. It may be too early to say how students and teachers will cope with online learning as they figure out the constraints and reorient to address them, but the perception of students is an important consideration, which we have tried to document.

Based on the findings of the study, we conclude that a majority of the students have accepted and found online learning to be a suitable method of education during the current pandemic. We found that they considered online learning advantageous due to the convenience and flexibility of this method with respect to time and place. Students preferred well-structured content with recorded videos uploaded in university websites. They also indicated the need for interactive sessions with quizzes and assignments at the end of each class to optimize the learning experience. Therefore, we can say that our study supports the findings of the study conducted by Abrami et al. in 2011 and Dhawan in 2020. However, not every student can accept and adapt to this change in the method of learning (Armstrong, 2011). Many students even claimed that a virtual mode of learning could be difficult in comparison to offline classroom learning environments due to a few hindrances such as practical demonstrations, the ineptitude of the instructor in operating ICT, and technical constraints (Koohang et al., 2014). Hence, to make online learning more effective, these factors must be considered while developing content. Once the pandemic is over, there may be growth of the online system in the educational sector. Furthermore, it was discerned in the current study that the majority of students opined that e-learning is a very innovative system for acquiring knowledge and skills (Floyd et al., 2003).

The present research study also reveals that online classes will be very interesting to students as well as educators [39]. Therefore, they emphasized their satisfaction with the adoption of the online learning system and their interaction with educators/teachers during online teaching and learning. Thus, it can be seen that the majority of learners have the potentiality to leverage an online learning mode as they find it easy to use. This study showed that online learning is a potential platform for use in the near future. Though proper transformation to online education is complex, the significance of using online learning cannot be avoided (Larreamendy-Joerns and Leinhardt, 2006). Hence, this study will prove useful for reimagining and redesigning higher education with components involving an online mode. It is nevertheless true that the results of this study are authentic to those educational institutions that rely heavily on the findings of online surveys. As this research study was conducted during the pandemic, there were no options to conduct an offline survey, and this study therefore overlooked students or learners who have little knowledge about e-learning platforms/resources [40]. In a nutshell, we can say that the results of this study will be helpful for policymakers and educational institutes to bring about some changes and improvements to the online learning methodology.

### Limitation of the Study

An online survey was conducted for the present study. Due to the adoption of web-based surveys during the ongoing pandemic, the questionnaire was only administered to students/learners who were using online platforms. It was difficult for the researchers to conduct an offline survey since the pandemic was ongoing. Consequently, it discouraged the participation of those students who are not/less attracted to IT environments and did not participate in the survey. However, these are the most disadvantaged students in terms of online education.

## 5. Conclusions

The main drawback of online classes is network connectivity. The government should take steps to increase broadband services in rural areas. Students are worried about COVID-19. Colleges and universities should provide counselling to students and increase their awareness about COVID-19, thus increasing their confidence to deal with it. Learning is a continuous and ever-evolving process. The educational institutions from schools to universities should consider the present crisis a blessing and use it as motivation to make digital education a major part of the future learning process for all learners. To build young minds in the present crisis, effective and well-rounded educational practice is needed. This will help students to improve the skills needed to drive their productivity, employability, health, and well-being in the decades to come and ensure the overall progress of Saudi Arabia.

## Figures and Tables

**Table 1:** Background information of the sample respondents (N = 294).

Basis	Categories	Frequency	Percentage
**Gender**	Male	172	58.5
	Female	122	41.5
**Age Group**	Up to 30 years	221	75.2
	31–50 years	71	24.1
	Above 50 years	2	0.7
**Academic Courses**	IT (IT, Computer Science, Statistics, and Mathematics)	7	2.4
	Engineering (Agricultural, Mechanical, Civil, and Electrical)	10	3.4
	Business (Accounting, Sectorial, Supply Chain and Marketing)	258	87.7
	Art and Design (Fashion and Industrial Art)	19	6.5
**Academic Year of Study**	1st year	56	19.0
	2nd year	97	33.0
	3rd year	93	31.6
	4th year	48	16.3
**Current Status**	On study leave	21	7.1
	Full time student	136	46.3
	Working and studying at the same time	137	46.6
**Sources of Data Collection**	Saudi Electronic University	53	18.0
	King Saud University	35	11.9
	Alfaisal University	30	10.2
	King Abdulaziz University	32	10.9
	Dar Al Uloom University	40	13.6
	Prince Sultan University	39	13.3
	Shaqra University	34	11.6
	Al Yamamah University	31	10.5

**Table 2:** Reliability analysis.

Construct	α	*f*
Benefits of Online Learning	0.943	10
Students’ Perceptions of Online Learning	0.968	14
Attitude of Students toward Online Learning	0.807	06

**Table 3:** KMO and Bartlett’s test.

Construct	No. of Items	Kaiser–Meyer–Olkin (KMO) Measure of Sampling Adequacy	Bartlett’s Test of Sphericity		
			Approx. Chi-Square (*χ*^2^)	df	Sig.
**Students’ Perceptions of Online Learning**	**14**	0.959	4268.837	91	0.000

**Table 4:** Rotated component matrix.

Code	Statements	Factor Loading (λ)			
		Factor-1 (Perceived Usefulness of E-Learning)	Factor-2 (Perceived Self-Efficacy of Using E-Learning)	Factor-3 (Perceived Ease of Use of E-Learning)	Factor-4 (Behavioral Intention of Using E-Learning)
UEL1	Learning through online mode provides the flexibility to study at a convenient time for the student.	0.816	__	__	__
UEL2	Online learning can allow students to learn regardless of the place.	0.779	__	__	__
UEL3	Many technologies facilitate online tests and submission of assignments.	0.884	__	__	__
UEL4	Interactive communication between learner and instructor without meeting is possible with the help of various electronic devices.	0.788	__	__	__
SEL5	I experience confidence while using e-learning mode.	__	0.754	__	__
SEL6	I feel self-assured while operating e-learning functions.	__	0.708	__	__
SEL7	Use of online learning content makes me feel confident.	__	0.702	__	__
EEL8	I believe e-learning platforms are user friendly.	__	__	0.801	__
EEL9	Use of e-learning mode to get necessary information is easier for me.	__	__	0.781	__
EEL10	I am optimistic to the use of e-learning services; it can make learning easy.	__	__	0.758	__
EEL11	There is compatibility between the e-learning setup and my way of learning.	__	__	0.801	__
IEL12	I will use e-learning to boost my knowledge.	__	__	__	0.894
IEL13	I intend to use e-learning to update my subject knowledge with the latest amendments.	__	__	__	0.891
IEL14	I intend to use e-learning as an autonomous (free) learning tool.	__	__	__	0.703

**Table 5:** Model fit analysis.

Name of Category	Required Fit Indices	Acceptable Limits	Obtained Values
Absolute Fit Indices	χ^2^	*p* > 0.05	0.162
	RMSEA	<0.05	0.039
	SRMR	<0.09	0.054
	GFI	>0.90	0.907
Incremental Fit Indices	AGFI	>0.80	0.866
	CFI	>0.90	0.969
	TLI	>0.90	0.962
	NFI	>0.90	0.953
Parsimonious Fit Index	CMIN/DF	<3	2.791

**Table 6:** Analysis summary of scalar estimates.

Path	Std. Regression Weights	Squared Multiple Correlations	Critical Ratio	*p*-Value
SPOL → PUEL	0.922	0.850	16.411	<0.001
SPOL → PSEL	0.887	0.787	14.569	<0.001
SPOL → PEEL	0.993	0.985	18.977	<0.001
SPOL → BIEL	0.943	0.890	20.772	<0.001
PUEL → UEL1	0.795	0.633	17.662	<0.001
PUEL → UEL2	0.810	0.656	15.443	<0.001
PUEL → UEL3	0.842	0.709	16.251	<0.001
PUEL → UEL4	0.841	0.707	16.219	<0.001
PSEL → SEL5	0.929	0.863	15.572	<0.001
PSEL → SEL6	0.935	0.875	30.694	<0.001
PSEL → SEL7	0.895	0.802	26.610	<0.001
PEEL → EEL8	0.849	0.722	19.421	<0.001
PEEL → EEL9	0.897	0.805	24.371	<0.001
PEEL → EEL10	0.855	0.730	21.600	<0.001
PEEL → EEL11	0.859	0.738	21.888	<0.001
BIEL → IEL12	0.888	0.789	18.769	<0.001
BIEL → IEL13	0.906	0.821	23.138	<0.001
BIEL → IEL14	0.852	0.726	20.320	<0.001

**Table 7:** Construct validity results.

Construct	Composite Reliability (CR)	Average Variance Extracted (AVE)
Apprehend Usefulness of E-learning	0.894	0.680
Apprehend Self-Efficacy of Using E-learning	0.942	0.843
Apprehend Ease of Use of E-learning	0.898	0.748
Behavioral Intention of Using E-learning	0.914	0.780

**Table 8:** Students’ Perceptions of Online Learning (SPOL).

Code	Statements	Mean	Standard Deviation
**I. Perceived Usefulness of E-learning (PUEL)**			
**UEL1**	Learning through online mode provides the flexibility to the study and convenient time to the student.	3.68	0.869
**UEL2**	Online learning can allow students to learn regardless of the place.	3.99	1.065
**UEL3**	Many technologies facilitate online tests and submission of assignments.	3.96	0.778
**UEL4**	Interactive communication between learner and instructor without meeting is possible with the help of various electronic devices.	3.85	0.992
**II. Perceived Self-Efficacy of Using E-learning (PSEL)**			
**SEL5**	I experience confidence while using e-learning mode.	3.66	0.648
**SEL6**	I feel self-assured while operating e-learning functions.	3.70	1.129
**SEL7**	Use of online learning content makes me feel confident.	3.73	0.983
**III. Perceived Ease of Use of E-learning (PEEL)**			
**EEL8**	I believe e-learning platforms are user friendly.	3.68	0.120
**EEL9**	Use of e-learning mode to get necessary information is easier for me.	3.83	1.097
**EEL10**	I am optimistic to the use of e-learning services; it can make learning easy.	3.59	0.182
**EEL11**	There is compatibility between the e-learning setup and my way of learning.	3.48	0.132
**IV. Behavioral Intention of Using E-learning (BIEL)**			
**IEL12**	I will use e-learning to boost my knowledge.	3.60	0.145
**IEL13**	I intend to use e-learning to update my subject knowledge with the latest amendments.	3.77	0.125
**IEL14**	I intend to use e-learning as an autonomous (free) learning tool.	3.77	0.134

**Table 9:** Benefits of online learning (BOL).

Code	Statements	Mean	Standard Deviation
BOL1	Flexibility in time and place.	3.78	0.841
BOL2	Ease and quick share of educational material.	3.95	0.782
BOL3	Improved collaboration and interactivity among students.	3.20	1.214
BOL4	Access to higher education for all applicants.	3.52	1.230
BOL5	Possibility of working with e-learning.	3.72	0.146
BOL6	Accommodates different types of learning styles.	3.60	1.062
BOL7	Quick feedback.	3.66	0.739
BOL8	Wide and diverse interactions.	3.56	0.568
BOL9	Access study resources effectively.	3.72	0.954
BOL10	Updated learning material.	3.83	0.867

**Table 10:** Attitudes of students toward online learning.

Code	Variables	SD (1)	D (2)	Total (1 + 2)	N (3)	A (4)	SA (5)	Total (4 + 5)
ASEL1	I like the idea of online learning.	8.5%	7.8%	16.3%	19.0%	31.7%	33.0%	64.7%
ASEL2	I think online learning is an innovative concept and must be encouraged.	6.1%	7.1%	13.2%	18.7%	39.2%	28.9%	68.1%
ASEL3	I think online learning platform will be fun to use.	6.5%	11.2%	17.7%	25.9%	34.0%	22.4%	56.4%
ASEL4	I do not believe e-learning is suitable for courses that need practical demonstrations.	7.8%	9.2%	17.0%	24.1%	32.4%	26.5%	58.9%
ASEL5	Employers will not give the same preference to e-learning graduates rather they will give preference to people who have conventional classroom learning.	6.5%	13.9%	20.4%	23.8%	33.0%	22.8%	55.8%
ASEL6	Classroom learning is more expensive than e-learning.	7.8%	10.5%	18.3%	24.1%	33.0%	24.6%	57.6%

## Data Availability

The data used to support the findings of this study are available from the corresponding authors upon request.
